# Auxin signaling and vascular cambium formation enable storage metabolism in cassava tuberous roots

**DOI:** 10.1093/jxb/erab106

**Published:** 2021-03-13

**Authors:** David Rüscher, José María Corral, Anna Vittoria Carluccio, Patrick A W Klemens, Andreas Gisel, Livia Stavolone, H Ekkehard Neuhaus, Frank Ludewig, Uwe Sonnewald, Wolfgang Zierer

**Affiliations:** 1 Friedrich-Alexander-University Erlangen-Nuremberg, Department of Biology, Division of Biochemistry, Staudtstrasse 5, Erlangen, Germany; 2 International Institute for Tropical Agriculture, Ibadan, Oyo State, Nigeria; 3 Institute for Sustainable Plant Protection, CNR, Bari, Italy; 4 Technical University Kaiserslautern, Department of Biology, Division of Plant Physiology, Erwin-Schrödinger-Str. 22, Kaiserslautern, Germany; 5 Institute for Biomedical Technologies, CNR, Bari, Italy; 6 MPI of Molecular Plant Physiology, Germany

**Keywords:** Auxin, cassava, development, gibberellin, parenchyma, root, starch, storage, transcriptomics, xylem

## Abstract

Cassava storage roots are among the most important root crops worldwide, and represent one of the most consumed staple foods in sub-Saharan Africa. The vegetatively propagated tropical shrub can form many starchy tuberous roots from its stem. These storage roots are formed through the activation of secondary root growth processes. However, the underlying genetic regulation of storage root development is largely unknown. Here we report distinct structural and transcriptional changes occurring during the early phases of storage root development. A pronounced increase in auxin-related transcripts and the transcriptional activation of secondary growth factors, as well as a decrease in gibberellin-related transcripts were observed during the early stages of secondary root growth. This was accompanied by increased cell wall biosynthesis, most notably increased during the initial xylem expansion within the root vasculature. Starch storage metabolism was activated only after the formation of the vascular cambium. The formation of non-lignified xylem parenchyma cells and the activation of starch storage metabolism coincided with increased expression of the *KNOX/BEL* genes *KNAT1*, *PENNYWISE*, and *POUND-FOOLISH*, indicating their importance for proper xylem parenchyma function.

## Teaser

Auxin-mediated activation of secondary growth and subsequent *KNOX/BEL* expression coincide with active storage metabolism in xylem parenchyma cells of the cassava tuberous root.

## Introduction

Cassava (*Manihot esculenta*) is an agronomically important root crop species grown in tropical and subtropical regions of the world (Carvalho *et al.*, 2018). Over 60% of the global annual cassava yield is produced in sub-Saharan Africa (http://faostat3.fao.org/), despite the crop being almost exclusively grown by smallholder farmers with limited resources. While grown on a large total field area, the yield per hectare is often low due to suboptimal agronomic practice, the lack of fertilizer, and/or high pathogen incidence. In addition, progress in cassava breeding lags behind that of other crops such as maize, wheat, or rice. Current cassava breeding focuses mostly on resistance and complex yield traits, such as dry matter content and root yield. A better understanding of cassava storage root development could facilitate breeding or biotechnological efforts aimed at improved storage root yield.

Cassava is clonally propagated through the planting of stem cuttings from which new shoots and roots can emerge. Cassava storage roots develop from stem-derived roots through the formation of a vascular cambium and subsequent secondary root growth (Chaweewan and Taylor, 2015; Mehdi *et al.*, 2019). The molecular mechanisms of secondary growth have mainly been studied in the model systems *Arabidopsis thaliana* and poplar (*Populus trichocarpa*), and, while many details are still elusive, much has been learned in the recent past (Fischer *et al.*, 2019). The vascular cambium develops from a procambium in between primary phloem and xylem tissue, and controls the formation of secondary phloem and xylem (Ragni and Greb, 2018; Fischer *et al.*, 2019). The initiation and maintenance of meristematic stem cells is largely regulated by the phytohormone auxin (Snow, 1935; Björklund *et al.*, 2007; Agusti *et al.*, 2011). Auxin is synthesized in the shoot apex and moves into the root via a polar transport mechanism governed by auxin influx (AUX) and efflux carriers (PIN) (Bennett *et al.*, 1996; Carraro *et al.*, 2012; Robert *et al.*, 2015). Here it leads to the formation of a stem cell organizer via CLASS III HOMEODOMAIN LEUCINE ZIPPER (HD-ZIPIII) transcription factor activity that regulates the position and maintenance of the vascular cambium (Smetana *et al.*, 2019). Auxin is not the only phytohormone involved in vascular cambium development. Cytokinin and gibberellin (GA) have a complex interplay with auxin in the meristem of poplar stems (Immanen *et al.*, 2016). Cytokinin plays a major role in defining cell identities and controlling cell division, while GA promotes cell elongation and the deposition of secondary cell walls and lignification (Eriksson *et al.*, 2000; Biemelt *et al.*, 2004; Fischer *et al.*, 2019).

While some studies have recently indicated the importance of early auxin signaling events, as well as reduced GA signaling during storage root formation in sweet potato (Noh *et al.*, 2010; Firon *et al.*, 2013; Singh *et al.*, 2019), less is known about storage root initiation in cassava. The importance of auxin and GA, as well as other phytohormones, could be shown for *in vitro* root formation (Sojikul *et al.*, 2015; Utsumi *et al.*, 2020). In addition, Ding *et al.* (2020) recently analyzed the impact of transcriptional and post-transcriptional regulation of starch and cell wall metabolism as well as hormone response genes in already established cassava storage roots. However, processes during the early stages of storage root development remain to be elucidated in cassava.

In this study, we focused on the morphological and transcriptional changes occurring during the early stages of storage root development under field and controlled greenhouse conditions. The transition of stem-derived roots towards starch-storing tuberous roots was analyzed via RNA sequencing (RNA-seq) and quantitative real-time PCR (qRT-PCR), as well as detailed histological studies. We identified pronounced auxin signaling events and the activation of secondary growth factors, as well as decreased GA signaling in the transition stages. This was accompanied by extensive regulation of cell wall biosynthesis genes, which probably is the result of the initial xylem expansion occurring during secondary growth within the root vasculature. After establishment of a vascular cambium, increased amounts of non-lignified parenchyma cells were observed. Starch storage was initiated in parenchyma cells and the corresponding samples displayed the transcriptional profiles of a starch storage crop. Interestingly, the activation of starch storage metabolism coincided with increased expression of the *KNOX/BEL* genes *KNOTTED-LIKE FROM ARABIDOPSIS THALIANA *(*KNAT1*)**, *PENNYWISE* (*PNY*) and *POUND-FOOLISH* (*PNF*), indicating their importance for proper xylem parenchyma function. Our study provides insight into the formation of the cassava vascular cambium and secondary growth, and provides a framework for subsequent genetic studies.

## Materials and methods

### Planting material and growth conditions

Cassava stakes of genotype TME419 were planted in a field at IITA Ibadan, Nigeria towards the end of the rainy season. Root samples were taken from three individual stakes and frozen in liquid nitrogen at 30, 38, and 60 days after planting (dap). The samples were used for transcriptome analysis. Cassava stakes of genotype TME7 were grown in a greenhouse in Erlangen, Germany under a light regime of 12 h light and 12 h dark. Temperature was kept at a constant 30 °C and 60% relative humidity. Two nodal-derived root samples were taken from four stakes each at 22, 26, 30, 34, 38, 42, and 60 dap. Approximately 5 mm root pieces of the primary bulking area at the proximal end of the root were stored in 70% ethanol for subsequent microscopy. Root tips were cut off and the root was frozen in liquid nitrogen. These samples were used for qRT-PCR.

### Determination of soluble sugars, starch, and free amino acids

Soluble sugars, starch, and amino acids were measured as described previously (Obata *et al.*, 2020).

### Histology and microscopy

Histology and microscopy was performed as described previously (Mehdi *et al.*, 2019).

### RNA extraction, RNA sequencing, and qRT-PCR

Total RNA was extracted from TME419 roots by combining a modified cetyltrimethylammonium bromide (CTAB)-based extraction method (Li *et al.*, 2008) with subsequent spin-column purification. Approximately 500 mg of sample material was ground in liquid nitrogen and mixed with 1 ml of pre-heated CTAB extraction buffer (2% CTAB, 2% PVP-40, 20 mM Tris–HCl, pH 8.0, 1.4 M NaCl, 20 mM EDTA). Samples were incubated at 65 °C for 15 min and centrifuged at 15 000 rpm at 4 °C for 5 min. The supernatant was transferred and mixed with an equal volume of cold chloroform:isoamyl alcohol (24:1) before centrifugation at 15 000 rpm for 10 min. The supernatant was mixed with 0.6 vol. of cold isopropanol and centrifuged at a maximum speed for 20 min. The pellet was washed with 70% ethanol, air-dried, and dissolved in nuclease-free water. After DNase I treatment, the resulting RNA was cleaned up using the kit RNA clean & concentrator™ (Zymo Research, USA) according to the manufacturer’s instructions.

RNA samples were depleted of rRNA (Ribo-Zero rRNA Removal Kit Plant, Illumina) and sequenced with Illumina technology to obtain an average of 20 million paired-end reads. Raw files contained between 21 million and 60 million paired-end reads.

RNA extraction of TME7 roots was performed using the Spectrum Plant Total RNA Kit (Sigma-Aldrich, St. Louis, MO, USA). cDNA was generated from 0.5 µg of RNA using RevertAid H Minus Reverse Transcriptase as indicated by the manufacturer (Thermo Fisher Scientific, Waltham, MA, USA). The cDNA diluted was 1:10 and quantification of gene expression was examined using GoTaq® qPCR Master Mix (Promega, Madison, WI, USA). The assay was mixed in a 96-well plate and measured in an AriaMx Real-time PCR System (Agilent, Santa Clara, CA, USA). The results were analyzed using the 2^–ΔΔCt^ method (Livak and Schmittgen, 2001).

### Read trimming and mapping

FastQ files containing the raw sequencing reads were quality checked using FastQC (v. 0.11.5; http://www.bioinformatics.babraham.ac.uk/projects/fastqc/) and MultiQC (v. 1.8; https://multiqc.info/). Adapter and quality trimming was performed in two steps utilizing the k-mer trimming tool BBduk (v. 38.96; https://sourceforge.net/projects/bbmap/) with its provided adapter sequences. A k-mer length of 21 was set, allowing a minimum k-mer length of 11 and two mismatches. Reads <35 nucleotides or an average quality <20 were excised, as well as individual bases below a quality of 20 at the ends of the read. The resulting FastQ files were mapped to the *M. esculenta* genome (v.7.1; https://genome.jgi.doe.gov/portal/pages/dynamicOrganismDownload.jsf?organism=Mesculenta) in two passes using STAR (v.2.5.0a; Dobin *et al.*, 2012; https://github.com/alexdobin/STAR). The resulting BAM files were indexed and deduplicated employing Samtools (v.1.7; Li *et al.*, 2009; http://www.htslib.org/). Read counting was performed using the program featureCounts (v.1.5.0; Liao *et al.*, 2013; http://bioinf.wehi.edu.au/featureCounts/). Only primary reads were counted. Trimmed, mapped, and deduplicated read counts are available in Table S1 at Dryad Digital Repository (https://doi.org/10.5061/dryad.0cfxpnw0t). All aforementioned programs were used under Linux (Ubuntu v. 18.04 LTS).

### Data analyses

Log2 fold change (log2FC) and its standard error were estimated in R (v. 3.6.2) utilizing the Bioconductor package DESeq2 (https://bioconductor.org/packages/release/bioc/html/DESeq2.html; Love *et al.*, 2014) on individual pairs. Wald’s test was used to calculate *P*-values between pairs, which were adjusted after Bonferroni’s family-wise error rate (FWER). Genes with |log2FC| ≥1 and FWER ≤0.05 were accepted as differentially expressed genes (DEGs). Enrichment analyses were conducted with a one-sided Fisher’s exact test using the Bioconductor package clusterProfiler. (https://bioconductor.org/packages/release/bioc/html/clusterProfiler.html; Yu *et al*.. 2012). Enrichments with FWER ≤0.05 were accepted as significant. Kyoto Encyclopedia of Genes and Genomes (KEGG) orthology (KO) terms, and cassava and thale cress identifiers were taken from an annotation file published with the genome. Pathway and regulatory networks were constructed through publication and database mining [STRING (https://string-db.org/), BioGRID (https://thebiogrid.org/), and TAIR (https://www.arabidopsis.org/)]. In the text, cassava genes were described by their best *A. thaliana* hit based on BLASTP similarity.

## Results and discussion

### Phenotypic and metabolic characterization of cassava root growth in the field

**Fig. 1.**
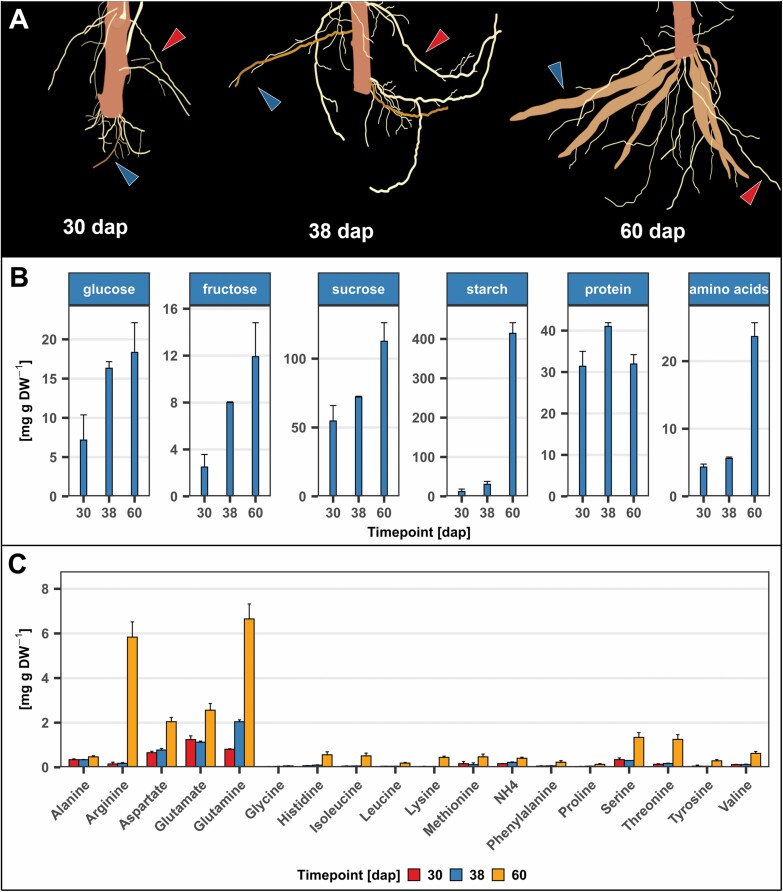
Phenotypic and metabolic characterization of root growth. (A) Schematic depiction of root phenotypes at sampling time points 30, 38, and 60 dap. Blue arrows indicate developing storage roots (DSRs) and red arrows indicate non-bulked roots (NBRs). (B) Glucose, fructose, sucrose, starch, protein, and total amino acid content of roots identified as (potential) storage roots. (C) Individual amino acids measured at different time points.

Stakes of cassava genotype TME419 were planted in a field in Ibadan, Nigeria for up to 60 d to evaluate cassava storage root growth. The plants were carefully dug up and sampled at 30, 38, and 60 dap. Two types of roots were observed during the experiment, one of which progressed towards storage roots. These root types were subsequently called non-bulked roots (NBRs) and developing storage roots (DSRs). While the two root types were difficult to distinguish at 30 dap, they became distinct due to increased stiffness and darkening around 38 dap (Fig. 1A). This color change is likely to be the result of formation of the root periderm, a protective tissue layer that contains an enrichment of phenolic compounds (Campilho *et al.*, 2020). The periderm is formed during secondary growth, making this root color change indicative for this process. Plants analyzed at 60 dap had clearly distintuishable tuberous roots that had already bulked considerably (Fig. 1A). Potential and visually distinguishable DSRs were analyzed for their soluble sugars, starch, proteins, and amino acids on a dry matter basis (Fig. 1B).

Soluble sugar concentrations increased linearly over time, with sucrose showing the highest abundance in comparison with glucose and fructose, indicating increased sucrose delivery and cleavage in storage roots. While there is a slight increase in starch concentration between 30 and 38 dap, an exponentially higher accumulation is observed between 38 and 60 dap. This is accompanied by a profound increase in free total amino acid concentrations. However, no elevation in protein concentration was observed. The high amino acid level at 60 dap was mainly due to accumulation of nitrogen-rich amino acids glutamine, glutamate, aspartate, and especially arginine, indicating increased delivery of nitrogen compounds from source tissues into storage roots (Fig. 1C). In contrast to many other root and tuber crops, cassava does not produce high abundant storage proteins (Vanderschuren *et al.*, 2014), which could explain the high levels of free nitrogen-rich amino acids.

Together, the phenotypic and metabolic data suggested the onset of secondary root growth between 30 and 38 dap, and the onset of storage metabolism in the tuberous root between 38 and 60 dap.

### Transcriptome analysis on cassava root types

**Fig. 2.**
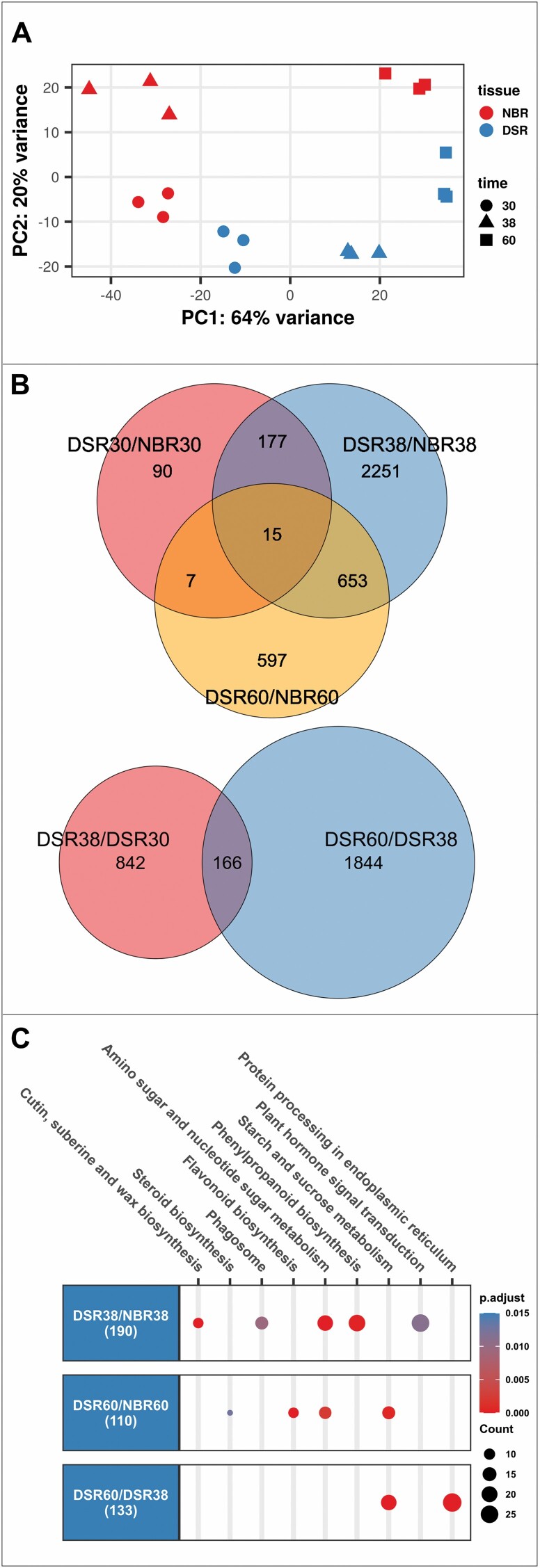
Basic characterization of the obtained cassava root transcriptomes. (A) Principal component analysis on non-bulked roots (NBRs) (red) and developing storage roots (DSRs) (blue) on log-transformed RNA-seq data at 30 (circle), 38 (triangle), and 60 dap (square). (B) Venn diagrams of differentially expressed genes (|log2FC| ≥1 and FWER ≤0.05) on pairwise comparisons. (C) KEGG pathway enrichment analysis on DEGs of selected pairs. Circles show the number of genes in a pathway, the color indicates FWER values, and the number in parentheses is the total number of genes with an annotated KO term within the comparison. Pathways with FWER ≤0.05 are shown.

To learn more about the underlying gene regulation of storage root formation, RNA-seq was performed on the different root samples. All replicates of each condition clustered together in a principal component anlysis (PCA) on log-transformed counts, demonstrating that no obvious sample outliers are present in the dataset (Fig. 2A). The different time points and tissues separated along PC1 and PC2, respectively, with PC1 accounting for 64% and PC2 for 20% of variance. This indicated a higher influence of time than tissue on the cassava root transcriptome. The sampled roots separated into two different root types, DSRs or NBRs, for the respective time points (DSR30, DSR38, DSR60, NBR30, NBR38, and NBR60). The analysis indicated that the sampled root types were rather similar on their transcriptional level at 30 dap but became more different at later stages (Fig. 2A). Only 90 of 3790 DEGs were specific for the time point 30 dap (Fig. 2B).

Most transcriptional changes were observed between the two root types at 38 dap, with >2000 genes being specifically regulated differently at this time point (Fig. 2B). This fitted the increasing visual distinction and onsetting secondary growth at 38 dap. Therefore, we focused our analysis on the remaining comparisons containing DSRs (DSR38/NBR38, DSR60/NBR60, DSR38/DSR30, and DSR60/DSR38). The up- and down-regulated genes were used for KEGG pathway enrichment (see Table S2 at Dryad).

This resulted in three significantly enriched pathways of particular interest: ‘Sucrose and starch metabolism’, ‘Amino sugar and nucleotide metabolism’, and ‘Plant hormone signal transduction’ in the significantly up-regulated genes of DSR38/NBR38, DSR60/NBR60, and DSR60/DSR38 (Fig. 2C).

The pathway ‘Sucrose and starch metabolism’ (see Table S3 at Dryad, sheet 1) was enriched in up-regulated genes of DSR60/NBR60 and DSR60/DSR38. Transcriptional up-regulation of starch biosynthesis during storage root development is expected considering the high starch content of cassava tuberous roots at 60 dap (Fig. 1B). The ‘Amino sugar and nucleotide sugar metabolism’ (Table S3 at Dryad, sheet 2) pathway was enriched in up-regulated genes of DSR38/NBR38 and DSR60/NBR60. This pathway included the biosynthesis of diffferent ADP-, GDP- and UDP-sugars necessary for primary and secondary cell wall synthesis, which had their highest epression in storage roots at 38 dap. The enrichment at the time point 60 dap was mainly caused by some overlapping genes with the ‘Sucrose and starch metabolism’ pathway, as well as different α-glucosidases and chitinases. Fewer cell wall metabolism genes were observed in DSR60/NBR60.

Genes contained in ‘Plant hormone signal transduction’ (see Table S3 at Dryad, sheet 3) were solely enriched in DSR38/NBR38, indicating that extensive developmental changes occurred around the 38 dap time point. Most notably, genes involved in auxin and cytokinin signaling were up-regulated in DSRs compared with NBRs. The significant enrichment of the ‘Phagosome’ (Table S3 at Dryad, sheet 4) in DSR38/NBR38 was caused by different *TUBULIN* and *RAC/RHO GTPase* genes. Although these gene groups have many different functions, their regulation might reflect a high activity of the cytoskeleton during periods of cell division and cellular restructuring.

### Regulation of starch and cell wall sugar biosynthesis

**Fig. 3.**
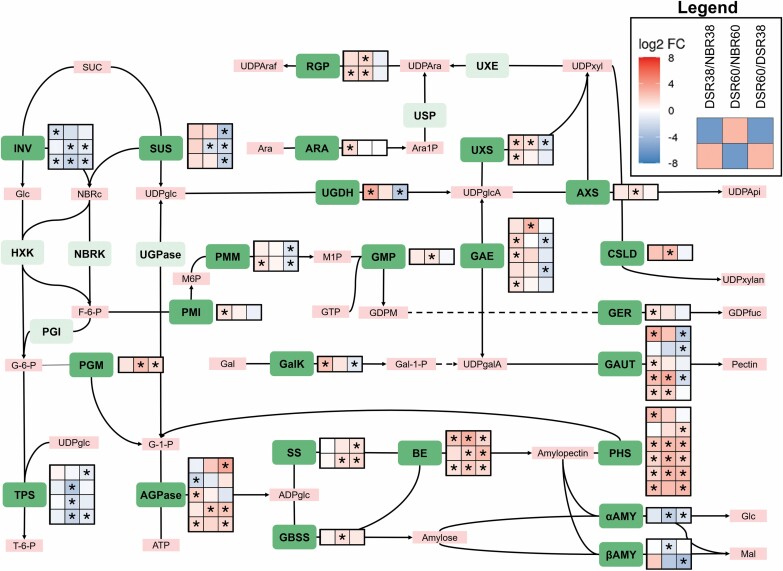
Overview of carbohydrate-related transcriptional changes between different root stages inferred from differentially expressed genes in the KEGG pathways ‘Amino sugar and nucleotide sugar metabolism’ and ‘Sucrose and starch metabolism’. Heat maps show the log2FC of genes that are differentially expressed in at least one comparison. Columns from left to right are DSR38/NBR38, DSR60/NBR60, and DSR60/DSR38. Asterisks indicate a significant difference in expression, with |log2FC| ≥1 and FWER ≤0.05. Green rectangles highlight genes with differential expression. Light colored rectangles represent players with no differentially expressed genes in expression. Red rectangles indicate metabolites.

Starch and cell wall biosynthesis both compete for monosaccharide building blocks. This was also reflected in the differential expression of genes involved in either pathway (Fig. 3). Genes involved in cell wall formation were generally up-regulated in DSR38/NBR38, while genes involved in starch synthesis were generally up-regulated in DSR60/NBR60.

UDP-Glc is a key substrate in the biosynthesis of most secondary cell wall sugars, especially in the form of UDP-GlcA. This interconversion is catalyzed by the enzyme UDP-glucose dehydrogenase (UGDH) (Tenhaken and Thulke, 1996). One *UGDH* gene (Manes.14G084200) showed the most characteristic expression pattern in relation to the KEGG pathway analysis (Fig. 2C) by being strongly up-regulated in DSR38/NBR38 and having next to no expression in DSR60. UGDH catalyzes the first non-reversible reaction in hemicellulose synthesis, and lack of the enzyme causes severe defects in secondary cell wall and pectin deposition as result of a low amount of UDP-GlcA (Reboul *et al.*, 2011). UDP-GlcA serves as a substrate for UDP-Xyl synthesis through the enzymes UDP-xylose synthase (UXS) or UDP-apiose/UDP-xylose synthase (AXS) (Kuang *et al.*, 2016). Two *UXS* genes (Manes.04G032900 and Manes.11G132500) were up-regulated at DSR38/NBR38 and either not up-regulated in DSR60/NBR60 or down-regulated in DSR60/DSR38. Xyl forms the backbone of xylans, the main polysaccharide in hemicellulose, whose biosynthesis is not yet fully understood (Pauly *et al.*, 2013). However, cellulose synthase-like D (CSLD) enzymes take part in this process (Bernal *et al.*, 2007). One *CSLD* gene (Manes.05G000900) was only up-regulated in DSR60/NBR60. These genes also play a role in correct meristem morphogenesis (Yang *et al.*, 2016). Plants use UDP-GlcA to produce the main pectin subunit, UDP-GalA, via the enzyme UDP-glucuronic acid epimerase (GAE) (Gu and Bar-Peled, 2004). Pectin itself is in part synthesized from UDP-GalA through galacturonosyl transferase (GAUT) enzymes (Atmodjo *et al.*, 2011). Five *GAUT* genes were differentially expressed and showed a similar trend to the other nucleotide-sugar biosynthesis genes (Manes.02G085400, Manes.03G122300, Manes.09G119300, Manes.14G088700, and Manes.15G076900). Pectins are part of the primary cell wall that exists in all cells. Generally speaking, nucleotide-sugar biosynthesis genes showed an up-regulation in DSR38/NBR38 and a down-regulation in DSR60/DSR38. This is especially true for genes directly using monosacharides UDP-Glc and fructose-6-phosphate. This gene expression pattern was probably the result of different cellular compositions of the different samples, with 38 dap samples containing a higher ratio of cells displaying a secondary cell wall and samples of 60 dap containing more parenchyma cells.

Genes involved in branching off sugars from sucrose breakdown towards secondary cell wall sugars were down-regulated again at stage DSR60 (Fig. 3). At this stage, starch biosynthesis genes were strongly expressed and were up-regulated between stage 38 and 60 dap (Fig. 3). This fits with the strong increase in starch measured at 60 dap compared with 38 dap (Fig. 1B). Notably, a plastidic *Phosphoglucomutase* (*PGM*) gene (Manes.14G031100) was strongly up-regulated in DSR60/DSR38 or DSR60/NBR60, indicating that more glucose-6-phosphate (G-6-P) was converted to glucose-1-phosphate (G-1-P) (Periappuram *et al.*, 2000) in established storage roots. In addition, one *Glucose-6-phosphate/phosphate translocator* (*GPT*; Manes.16G010700) was strongly up-regulated in the same way as *PGM*, indicating G-1-P production directly in amyloplasts. G-1-P is the substrate for ADP-glucose pyrophosphorylase (AGPase), which forms ADP-Glc, the substrate for starch biosynthesis. Five genes coding for AGPase subunits were differentially expressed during root bulking, of which two small subunits (Manes.12G067900 and Manes.18G019650) and one large subunit (Manes.11G085500) were up-regulated at DSR60/DSR38. The production of amylose is mainly catalyzed by the enzyme granule-bound starch synthase (GBSS) (Kuipers *et al.*, 1994). Cassava *GBSS* was up-regulated at DSR60/NBR60 with a non-significant up-regulation in DSR60/DSR38 (Manes.02G001000). Both differentially expressed STARCH SYNTHASE (*SS*) genes, one *SS1* (Manes.01G184000) and one *SS2* (Manes.02G046900), code for plastid-localized enzymes and were up-regulated in DSR60/DSR38. *SS1* was shown to be a major determinant of starch synthesis (Delvallé *et al.*, 2005). The other important enzyme in amylopectin synthesis, starch branching enzyme (BE) that produces the characteristic α-1-6-glucosyl branches (Guan *et al.*, 1995), also showed up-regulated genes in DSR60/NBR60 and DSR60/DSR38 (Manes.05G133800, Manes.08G022400, and Manes.S024960). Furthermore, starch phosphorylases (PHSs), that catalyze the reversible phosphorylation of starch to G-1-P (Hanes and Blackman, 1940), were up-regulated in a similar manner to the other starch genes. These consist of four plastidic (Manes.02G052280, Manes.02G052360, Manes.02G052520, and Manes.02G052600) and two cytosolic forms of *PHS* (Manes.02G122500 and Manes.17G049200). The presence of active *PHS* genes together with starch biosynthesis genes speaks for their role in starch remodeling rather than breakdown, especially because other starch catabolic genes, such as *α-amylases* (*αAMY*; Manes.18G037100) and *β-amylases* (*βAMY*; Manes.03G155800, Manes.12G078500, and Manes.15G060100) showed the opposite trend by being down-regulated in DSR60/DSR38 or DSR60/NBR60. The same trend was seen for four genes annotated as *Trehalose-6-phosphate synthase/phoSphatase* (*TPS/TPP*) genes (Manes.01G198900, Manes.05G087900, Manes.06G103000, and Manes.15G098800). Different TPS/TPP proteins can catalyze either the production of trehalose-6-phosphate (T-6-P) from UDP-Glc or the back reaction, while others are not catalytically active and only have a regulatory purpose (Fichtner *et al.*, 2020), making an interpretation of these DEGs difficult.

One *vacuolar invertase* (Manes.01G076500) and two *CELL WALL INVERTASE* (*cwINV*; Manes.03G049200 and Manes.11G025400) genes were down-regulated in a similar fashion. Reduction in *cwINV* expression fits the observation that cassava switches from an apoplasmic mode of transport in non-bulked roots to a symplasmic mode of transport in tuberous roots (Mehdi *et al.*, 2019). Tuberous roots were shown to possess high sucrose synthase (SUS) activity (Mehdi *et al.*, 2019). Indeed, the main *SUS* genes of tuberous roots (Manes.01G221900, Manes.03G044400, and Manes.16G090600) were highly expressed; however, their transcription was not significantly regulated between the different root samples. Three less expressed *SUS* genes, consisting of two *SUS5* genes (Manes.01G123800 and Manes. 02G081500) and one *SUS6* gene (Manes.14G107800), were down-regulated in DSR60/DSR38 and their proteins are only minor contibutors to the total SUS protein amount in tuberous roots (Mehdi *et al.*, 2019). Similar to SUS, UDP-glucose pyrophosphorylase (UGPase), the enzyme that interconverts UDP-Glc and G-1-P, was highly expressed but not significantly differently regulated between the samples. Transcriptional up-regulation of *SUS* and *UGPase* was demonstrated in very early stages of potato tuber formation (Zrenner *et al.*, 1993; Ferreira and Sonnewald, 2012), but could not be demonstrated for cassava tuberous roots in this study, indicating either an even earlier onset of *SUS* expression or post-transcriptional regulation.

### Auxin signaling and vascular cambium establishment

Secondary growth in roots is accomplished through asymmetric cell division of stem cells. Previous studies on the morphology of cassava plants showed that adult storage roots continuously grow from a single vascular cambium ring, which resembles the secondary growth of model plants such as *A. thaliana* or poplar.

**Fig. 4.**
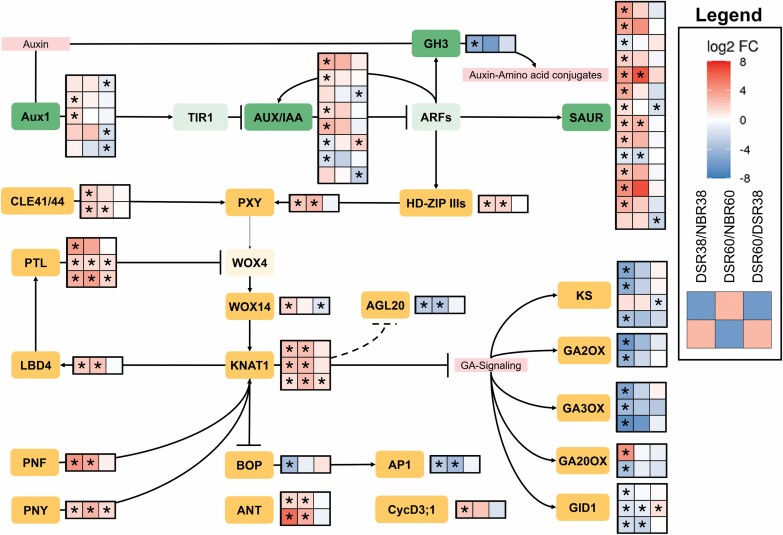
Overview on developmental-related changes between different root stages inferred from differentially expressed genes in the KEGG pathway ‘Plant hormone signal transduction’ (green) and from published regulations and interactions (orange). Heat maps show the log2FC of genes that are differentially expressed in at least one comparison. Columns from left to right are: DSR38/NBR38, DSR60/NBR60, and DSR60/DSR38. Asterisks indicate a significant difference in expression, with |log2FC| ≥1 and FWER ≤0.05. Light colored rectangles represent players with no differentially expressed genes in expression.

In Arabidopsis, the vascular cambium is induced by an initial auxin maximum causing the expression of *HD-ZIPIII* genes in procambium and pericycle cells that are connected to primary xylem cells (Smetana *et al.*, 2019). In this study, one *PHABULOSA* (*PHB*) gene (Manes.06G055000) was significantly up-regulated in DSR38/NBR38 and DSR60/NBR60 (Fig. 4). As expected, this coincides with an enrichment of hormone signal transduction (Fig. 2C) in DSR38/NBR38, of which most DEGs are part of the auxin signal transduction pathway (Fig. 4, green). These included two differentially expressed *AUXIN RESISTANT 1* (*AUX1*) genes (Manes.07G127400 and Manes.08G090700). *AUX1* encodes an auxin influx carrier and takes part in auxin transport into target tissues during gravitropism and root development (Yang *et al.*, 2006; Band *et al.*, 2014). After reception, auxin acts by inducing proteolysis of AUXIN RESISTANT/INDOLE-3-ACETIC ACID INDUCIBLE (AUX/IAA) proteins. This leads to the activation of AUXIN RESPONSE FACTORS (ARFs) at the protein level. Here, four *AUX/IAA* genes were up-regulated in DSR38/NBR38 (Manes.09G152700, Manes.11G051200, Manes.16G112900, and Manes.16G122601), while no ARFs showed differential expression between the two tissues. However, 11 *SMALL AUXIN UPREGULATED RNA* (*SAUR*) genes were up-regulated (Manes.04G080700, Manes.04G081500, Manes.04G082200, Manes.05G149166, Manes.05G149400, Manes.05G191200, Manes.09G064000, Manes.11G068170, Manes.11G091848, Manes.18G015150, and Manes.S086000). As the name suggests, *SAUR* genes are up-regulated upon auxin signaling and are commonly used as indicators for an active auxin response (McClure and Guilfoyle, 1987, 1989; Esmon *et al.*, 2006). One *GRETCHEN HAGEN 3/INDOLE-3-ACETIC ACID-AMIDO SYNTHASE* (*GH3.1*) gene was down-regulated in DSR38/NBR38. These genes are usually up-regulated in response to auxin. They catalyze the conjugation of indole-3-acetic acid with amino acids to inactivate the phytohormone. *Arabidopsis thaliana GH3.1* mutants show hypersensitive auxin responses (Staswick *et al.*, 2005).

Other genes that were expressed along with *PHB* included important genes during vascular tissue development such as the receptor gene *PHLOEM INTERCALATED WITH XYLEM* (*PXY*) (Manes.14G037000) and its ligands *CLAVATA3/ESR-RELATED 41* (*CLE41*) or *CLE44* (Manes.12G052002 and Manes.13G045100). In *A. thaliana* roots, *PXY* is controlled through *HD-ZIPIII* activity and, together with *CLE41/44*, retains stem cell activity in the vascular cambium (Fisher and Turner, 2007; Hirakawa *et al.*, 2008; Etchells and Turner, 2010; Smetana *et al.*, 2019) through *WUSCHEL-RELATED HOMEOBOX 4* (*WOX4*) and subsequent *WOX14* expression. (Hirakawa *et al.*, 2010; Etchells *et al.*, 2013). However, no *WOX4* gene was differentially expressed and no gene in the current annotation file was annotated as *WOX14*. Nevertheless, the closest *AtWOX14* homolog (Manes.05G124300) was significantly up-regulated in DSR38/NBR38 (see Fig. S1 at Dryad). Further important genes in vascular cambium establishment, maintenance, and regulation of differentiation are *KNOTTED-LIKE HOMEOBOX* (*KNOX*) and *BELLRINGER* (*BEL*). Three *KNAT1* genes in this dataset (Manes.05G184900, Manes.18G051700, and Manes.18G052901) were significantly up-regulated in DSR38/NBR38 and DSR60/NBR60, but showed much higher expression in DSR60 than in DSR38. KNOX proteins function as heterodimers together with BEL factors (Hay and Tsiantis, 2010). KNAT1 is known to interact with the functional paralogs PNY and PNF (Smith *et al.*, 2004; Ragni *et al.*, 2008), both of which have genes in cassava that showed the same expression pattern as *KNAT1* (Manes.09G097500 and Manes.11G148000). These interaction pairs are known to repress the expression of boundary genes *BLADE-ON-PETIOLE 1* (*BOP1*) or *BOP2*, as is the case here, with one *BOP2* (Manes.08G056500) gene being down-regulated in DSRs (Woerlen *et al.*, 2017). Despite this, the *BOP2* gene was most highly down-regulated at DSR38/NBR38 and the *KNAT1* genes had their highest expression at 60 dap. Downstream factors of *BOP2* showed a similar pattern to the boundary gene. These included a gene of the flowering gene *APETALA1/AGAMOUS-like 7* (*AP1/AGL7*; Manes.01G103200). Another flowering time gene, *AGL20* (Manes.05G041900), was down-regulated in a similar manner. Other crop species such as potatoes or onions utilize an adapted flowering pathway for storage organ initiation with genes of the *FLOWERING LOCUS T* (*FT*) serving as a mobile induction signal but also being expressed in the storage organ (Navarro *et al.*, 2011; Lee *et al.*, 2013; Abelenda *et al.*, 2014). No expression of *FT* genes was found in any samples. The cassava storage root is a perennial organ that is not linked to reproduction. This implies a different regulation in comparison with vegetative reproduction organs—potatoes or onion bulbs. In addition, lack of flowering time genes has been linked to perennial growth (Melzer *et al.*, 2008). *KNAT1* was associated with the expression of the boundary gene *lob domain-containing protein* (*LBD*) *4* (Zhang *et al.*, 2019). One gene was up-regulated in DSR38/NBR38 and DSR60/NBR60 (Manes.06G173400). *LBD4* takes part in keeping the procambium–phloem boundary together with *PXY* (Smit *et al.*, 2020) and is able to promote the expression of *PETAL LOSS* (*PTL*) on the phloem side of the vasculature where it restricts expression of *WOX4* to maintain organ boundaries (Zhang *et al.*, 2019). Three *PTL* genes were up-regulated in at least DSR38 versus NBR38 (Manes.10G055600), with two also being up-regulated at SR60/NBR60 and DSR60/DSR38 (Manes.07G000300 and Manes.08G021500).

Up-regulation of auxin-related transcripts seems to be paralleled by a general down-regulation of GA-related transcripts. Changes in GA regulation have been reported during storage organ formation in potato and sweetpotato (Rosin *et al.*, 2003; Singh *et al.*, 2019). With the exception of one up-regulated GA20 oxidase (Manes.S084560), all DEGs involved in GA metabolism are down-regulated in DSR38/NBR38 or over time in DSRs. This includes four *ENT-KAURENE SYNTHASE* (*KS*) genes (Manes.16G000900, Manes.16G047800, Manes.16G065800, and Manes.16G067901), three *GIBBERELLIN-2 OXIDASE* (*GA2OX*) genes (Manes.01G231100, Manes.15G121600, and Manes.17G070100), three *GIBBERELLIN-3 OXIDASE* (*GA3OX*) genes (Manes.04G120200, Manes.11G050500, and Manes.16G067700), and one *GIBBERELLIN-20 OXIDASE* (*GA20OX*) (Manes.05G042300) gene. While the regulation of GA-related genes is likely to be different depending on the cell type, an overall much lower expression was observed in DSRs compared with their non-bulked counterparts.

Finally, the cambium gene *AINTEGUMENTA* (*ANT*) had two genes up-regulated in DSR38/NBR38 and DSR60/NBR60 (Manes.05G184000 and Manes.18G050000). *ANT* controls cell proliferation and differentiation, with overexpression of the gene in *A. thaliana* causing higher cell number and larger organs without altering the shape of the plant (Mizukami and Fischer, 2000). *ANT* works together with the cell cycle control gene *Cyclin D3;1* (*CycD3;1*) in a cytokinin-dependent manner (Randall *et al.*, 2015). One *CycD3;1* gene (Manes.07G076800) was up-regulated in DSR38/NBR38. While there were no differentially expressed cytokinin biosynthesis genes in our data, some genes in cytokinin signal transduction are up-regulated in DSR38/NBR38 or DSR60/NBR60, namely *CYTOKININ RECEPTOR 1* like gene (*CRE1*; Manes.15G137400), *ARABIDOPSIS THALIANA RESPONSE REGULATOR 3* (*ARR3*; Manes.18G111900), and *ARABIDOPSIS THALIANA HISTIDINE PHOSPHOTRANSFER PROTEINS* (*AHP*; Manes.04G134100).

Overall, the results of the transcriptome analysis indicated that the DSRs underwent extensive cellular restructuring before the storage metabolism was activated. This can be derived from the strong transcriptional activation of genes encoding cell wall-modifying enzymes and hormone signaling components at stage 38 dap, as well as from the subsequent activation of storage metabolism between 38 and 60 dap. Although many of the developmental genes mentioned above were not significantly regulated within the comparisons of DSR samples, they showed differential expression between NBR and DSR samples, indicating that both tissues underwent a different program during their development. To gain a deeper insight into the underlying structural and transcriptional processes occurring in DSRs and to validate the field results independently, we performed a controlled greenhouse time-course experiment, covering the sampling time points of the RNA-seq experiment, as well as additional time points. The samples were used for histological analysis and qRT-PCR verification.

### Morphological changes during root bulking

**Fig. 5.**
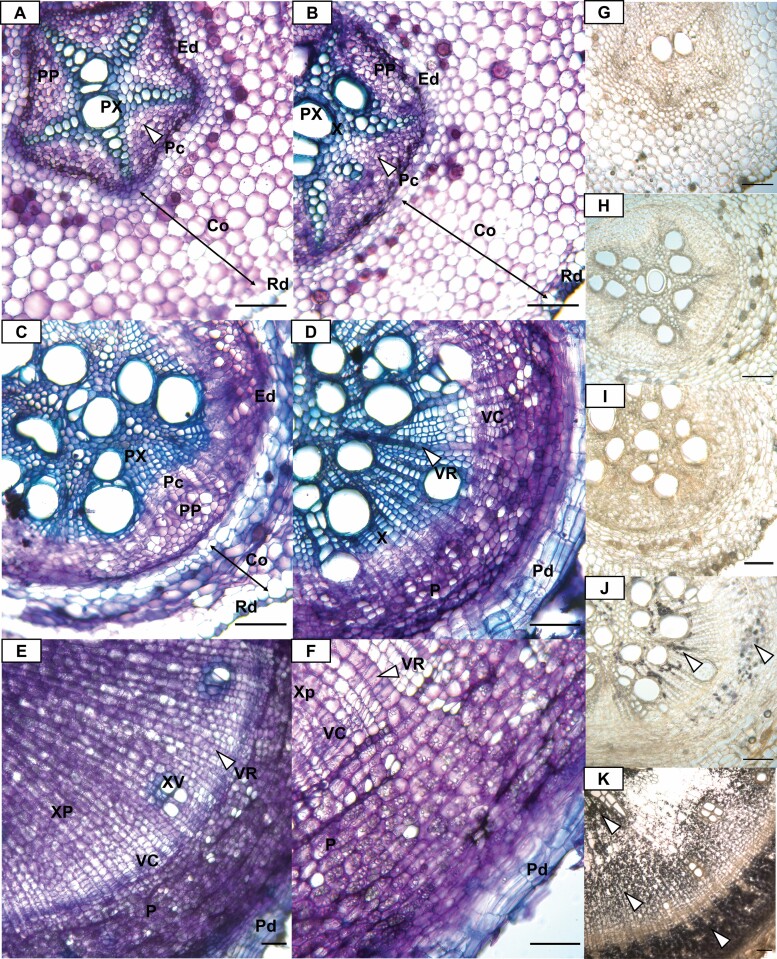
Histological analysis of cassava storage root development. (A–F) Toluidine blue-stained cross-section of different storage root stages. (A) Stage one roots show a star-shaped central cylinder enclosed by an endodermis. (B) Stage two roots have a ring-shaped central cylinder with ongoing cell division depositing xylem and phloem cells within the vasculature. The endodermis is still intact, and the cortex is still healthy. (C) Stage three root with a round central cylinder still enclosed in an intact endodermis. Xylem and phloem are irregularly shaped and cortex cells are severely squashed. (D) Stage four storage root with radial symmetry and no central cylinder. Rhizodermis is replaced with a periderm, and vascular ray cells are visible. Xylem displayed only lignified cells. (E and F) Stage five tuberous root with increased lateral size through deposition of xylem parenchyma cells through the vascular cambium. (G–K) Cross-section of storage root from stage one to five stained with Lugol solution. Scale bars indicate 100 µm. Abbreviations: Co, cortex; Ed, endodermis; PP, primary phloem; P, phloem; PX, primary xylem; Pc, procambium; Pd, periderm; Rd, rhizodermis; VC, vascular cambium; VR, vascular ray; X, xylem; XP, xylem parenchyma; XV, xylem vessel.

Stakes of cassava genotype TME7, a genotype closely related to TME419 (Bredeson *et al.*, 2016), were planted in the greenhouse. Potential and developing storage roots emerging from the nodal areas of cassava stem pieces were sampled at seven time points (22, 26, 30, 34, 38, 42, and 60 dap) to obtain a broad line-up of DSRs at different ages and developmental stages. Cross-sections of the primary bulking site of every sampled root were taken, and the resulting sections were stained with toluidine blue solution in order to be able to group these roots according to their morphology, rather than their sampling time point (Fig. 5A–F).

Developing roots were sorted into five developmental stages based on their cellular composition and appearance. Stage one roots had a typical primary root morphology (Fig. 5A) and were observed in plants at 22 or 26 dap. They contained a star-shaped central cylinder encased in an endodermis. Inside the central cylinder were alternating primary phloem and xylem cells with a procambium in between (indicated by a small layer of dividing cells).

The radial symmetry of the DSR was established between stages two and four (Fig. 5B–D). In stage two (Fig. 5B), the shape of the xylem and phloem tissue was less organized compared with stage one, indicating ongoing asymmetric cell division of the procambium that produces new phloem and xylem cells. The endodermis was pushed towards the outside, which led to a circular shape of the central cylinder. The cortex cells were still more or less intact. Stage three nodal roots showed a further increase in size of the central cylinder (Fig. 5C). The cambium was highly active and had a larger diameter than in the previous stage, albeit not yet being of radial shape. The newly formed cells were mostly lignified xylem cells, as indicated by the blue coloring of the newly formed cells towards the center. This fits the strong transcription activation of cell wall-related genes in field-grown plants at time point 38 dap. The phloem tissue did not increase much in comparison with younger roots. The endodermis was still intact, but the cortex was severely squashed towards the rhizodermis. The first vascular rays appeared but were hard to pinpoint at this stage. No starch was formed up to this point (Fig. 5G–I). Stage four (Fig. 5D) DSRs were marked by dispersal of the endodermis and an annular shape of the inner xylem, as well as an overall radial shape of the root. The cortex was completely missing and the rhizodermis was displaced by a periderm at this stage. The root had not yet grown in diameter. However, the periderm gave it a visible brown coloring compared with the pale white of young roots. Furthermore, starch storage began in this phase of root development as indicated by Lugol staining (Fig. 5J). The stain was visible in the inner xylem cells adjacent to the vascular ray and the ray cells themselves, but most of the starch was still stored in phloem parenchyma cells. Similar to the field samples (Fig. 1A), a strong increase in starch is only measurable in established storage roots at ~60 dap (see Fig. S2A at Dryad). The color change between stage three and four was the main factor in root sampling for the transcriptome experiment, making this the likely developmental range of DSRs at 38 dap. Transcriptional changes at this time point were predominantly characterized by higher expression of secondary cell wall synthesis genes in tuberous roots. Secondary cell walls are solely deposited in differentiated sclerenchyma cells (Zhong *et al.*, 2019). These include lignified xylem cells, which were strongly produced at this developmental stage (Fig. 5C, D). The number of meristematic cells increased, which explains the high expression of cambium genes, namely *PXY* (Etchells and Turner, 2010). Furthermore, the procambium developed into the ring-shaped vascular cambium during this time. This coincided with higher expression of auxin signal transduction genes and *HD-ZIPIII*.

The more established storage roots (stage five; Fig. 5E, F) were easily identified on a macroscopic level due to an increase in lateral size. The vascular cambium mostly produced xylem parenchyma cells that were rapidly filled with starch, which is indicated by even the youngest cells being stained by the Lugol solution (Fig. 5K). This coincided with down-regulation of secondary cell wall genes, up-regulation of starch biosynthesis genes, high amounts of starch (Figs 1B, 5K; see Fig. S2 at Dryad), as well as strong up-regulation of *KNOX* and *BEL* genes *KNAT1*, *PNY*, and *PNF.* In *A. thaliana*, *KNAT1* is able to regulate the differentiation of xylem fibers in secondary meristems (Byrne *et al.*, 2002; Mele *et al.*, 2003; Liebsch *et al.*, 2014) through regulation of GA signaling (Hay *et al.*, 2002; Bolduc and Hake, 2009; Felipo-Benavent *et al.*, 2018). In roots and hypocotyls of *A. thaliana*, *KNAT1* promotes differentiation (Liebsch *et al.*, 2014), while its overexpression in aerial parts of the plant leads to fewer lignified cells (Mele *et al.*, 2003), a function that is similar in poplar trees (Groover *et al.*, 2006). In other root and tuber crops, *KNOX/BEL* modules play a necessary role in the development of starchy storage organs. Sweet potatoes show similar expression levels of a *BEL1* gene in late developmental stages compared with *PNF* in this study (Dong *et al.*, 2019), which goes hand-in-hand with low GA signaling causing lower amounts of lignified cells to be produced (Singh *et al.*, 2019). In potatoes, the *KNOX* gene *POTATO HOMEOBOX 1* (*POTH1*) acts in a similar manner together with the BEL factor *BEL5* (Rosin *et al.*, 2003; Banerjee *et al.*, 2006; Lin *et al.*, 2013). Our study suggests that the low amount of lignified xylem cells was related to high *KNAT1*/*PNF* and/or *KNAT1*/*PNY* activity in cassava tuberous root after establishment of the vascular cambium.

### Independent qPCR validation of observed transcriptional changes

To validate and further elucidate the results of the RNA-seq analysis, a set of genes was chosen to represent the most important pathways and regulatory networks (Table 1; primer list in Table S4 at Dryad) found to be regulated during storage root development in the transcriptome analysis. The expression was analyzed via qRT-PCR on the different DSR stages. The respective morphology and corresponding root stage were microscopically confirmed for every root.

**Table 1.** Selected genes for qRT-PCR validation

Abbreviation	Name	Identifier
*AGL20*	*AGAMOUS-like 20*	Manes.01G263500
*ANT*	*AINTEGUMENTA*	Manes.05G184000
*AUX1*	*AUXIN RESISTANT 1*	Manes.07G127400
*BOP2*	*BLADE-ON-PETIOLE*	Manes.08G056500
*CLE41*	*CLAVATA3/ESR-RELATED 41*	Manes.13G045100
*GH3.1*	*GRETCHEN HAGEN 3.1/INDOLE-3-ACETIC ACID-AMIDO SYNTHASE*	Manes.13G116000
*GID1B*	*GIBBERELLIN INSENSITIVE DWARF 1B*	Manes.01G212300
*KNAT1*	*KNOTTED-LIKE FROM ARABIDOPSIS THALIANA 1*	Manes.05G184900
*LBD4*	*LOB DOMAIN-CONTAINING PROTEIN 4*	Manes.06G173400
*PMI*	*PHOSPHOMANNOSE ISOMERASE*	Manes.02G069000
*PGM*	*PHOSPHOGLUCOMUTASE*	Manes.14G031100
*PHB*	*PHABULOSA*	Manes.06G055000
*PHS*	*STARCH PHOSPHORYLASE*	Manes.02G052600
*PNF*	*POUND-FOOLISH*	Manes.11G148000
*PNY*	*PENNYWISE*	Manes.09G097500
*PXY*	*PHLOEM INTERCALATED WITH XYLEM*	Manes.14G037000
*TPS*	*TREHALOSE-6-PHOSPHATE SYNTHASE/PHOSPHATASE*	Manes.06G103000
*UGDH*	*UDP-GLUCOSE DEHYDROGENASE*	Manes.14G084200
*SUS*	*SUCROSE SYNTHASE*	Manes.03G044400
*UGPASE*	*UDP-GLUCOSE PYROPHOSPHORYLASE*	Manes.02G080600

**Fig. 6.**
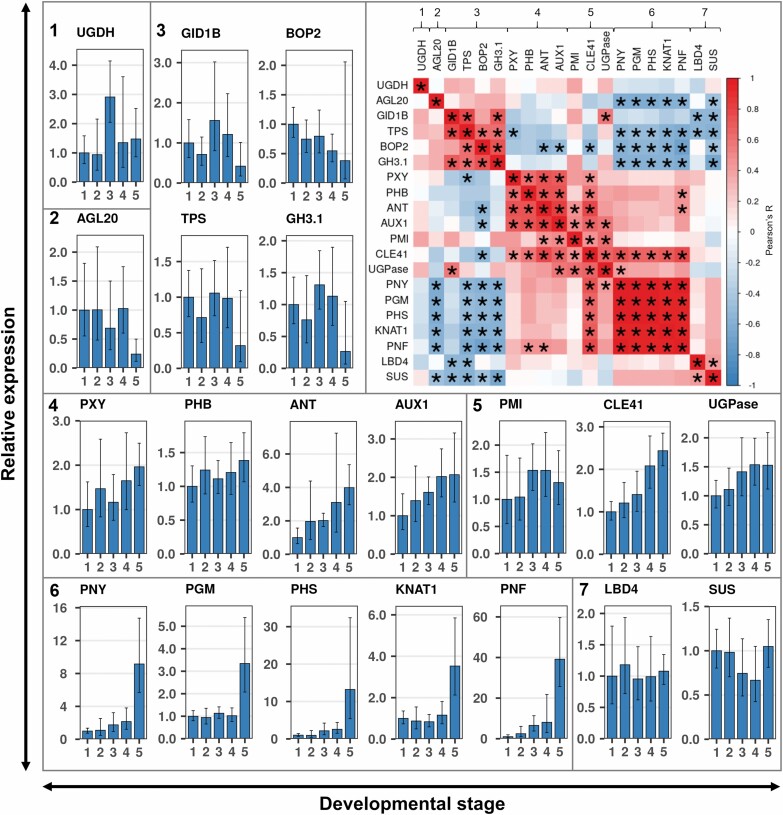
Quantitative RT-PCR validation of selected genes regulated during root development. Bars show the mean relative expression normalized to three housekeeping genes [Manes.06G116400 (*Glyceraldehyde 3-phosphate dehydrogenase/*GAPDH), Manes.06G058300 (*Histidinol dehydrogenase*/HDD), and Manes.09G039900 (*serine/threonine-protein phosphatase 2A catalytic subunit*/PP2A)] of developing storage roots (stages 1–5). Relative expression was calculated using the 2^–ΔΔCt^ method in relation to stage one. The *x*- and *y*-axes of the plots show developmental stage and relative expression, respectively. Gene names are given in the plot titles. The heatmap on the top right shows the Pearson’s *R* value of pairwise comparisons. Asterisks indicate significant correlation (FWER ≤0.05 as indicated by differences in asterisks). The genes were hierarchically clustered after Ward’s D2.

The *BEL* factor genes *PNY* and *PNF* were strongly up-regulated in stage five DSRs, with a slight upward trend from stage one to four (Fig. 6). *PNF* expression was especially high, with a 40-fold up-regulation in the more established DSRs in comparison with stage one. A very similar trend is shown in the expression of *KNAT1*. The expression of these three genes correlated with the expression of starch genes *PGM* and *PHS*, but also with the production of a high number of xylem parenchyma cells (Fig. 5 E, F, K).

The boundary genes behaved as predicted. *BOP2* expression showed a downward trend with especially low expression at stage five, which coincided with high expression of the *KNAT1/BEL* module. They also showed a significant negative correlation (Fig. 6, top left). *LBD4*, while not being up-regulated throughout root bulking, was expressed similarly to the transcriptome dataset at every stage.

*PXY* expression was detectable in every stage of root bulking. However, the expression dropped at stage three and went up again in later stages. This was highly correlated with the expression of *PHB*, which supports the hypothesis shown in Fig. 4 that *PXY* expression in cassava is dependent on an auxin signal inducing *PHB* expression, even though the change in the *HD-ZIPIII* gene is rather minute in comparison with *PXY* (Smetana *et al.*, 2019; Zhang *et al.*, 2019). This expression pattern was exactly contrary to the expression levels of *GID1B* (*GIBBERELLIN INSENSITIVE DWARF 1B*) and *TPS* as well as, to a lesser extent, *GH3.1*, *PMI (PHOSPHOMANNOSE ISOMERASE)*, and *BOP2*. This coincided with deposition of lignified xylem cells by the vascular cambium between stage three and four (Fig. 5C, D) and with a comparably small up-regulation of the GA receptor gene *GID1B*, which might indicate a short GA signaling spike for the production of lignified cells. The starch biosynthesis genes *PHS* and *PGM* were strongly expressed at stage five in DSRs and have close to no expression at the other stages. This fits the histological analysis, where starch was mainly produced at stage five with negligible amounts in stage four and no starch in stages one to three (Fig. 5G–L; see Fig. S2A at Dryad). Expression of these starch genes varied greatly within stage five roots but did correlate with the starch content of the particular roots (Fig. S2B, C at Dryad). *AGL20* is strongly down-regulated between stage four and five, showing strong opposite behavior to the *KNOX/BEL* and *PGM/PHS* genes. *ANT*, *CLE41*, and *AUX1* increased in expression with advancing development. Similar to the transcriptomic results, *SUS* and *UGPase* did not exceed a |log2FC| of 1 between the DSR stages.

The gene expression results obtained from independent samples with an independent method support the hypothesis formed through the transcriptome analysis, since the genes clustered in distinct groups (Fig. 6, top left) or otherwise resembled the transcriptional profile shown in Figs 3 and 4. *GID1B*, *TPS*, *BOP2*, and *GH3.1* formed a cluster as expected and were more highly expressed in early stages of nodal roots (Fig. 6, cluster 1). The proposed auxin signaling genes (*AUX1*, *PHB*, and *PXY*) were tightly correlated with each other, as well as with *ANT* (Fig. 6, cluster 2). Starch biosynthesis genes were only strongly expressed in stage five roots together with the *KNOX/BEL* genes (Fig. 6, cluster 6), in accordance with the transcriptome dataset.

### Conclusion

This study investigated the transcriptional and morphological changes occurring during early storage root development. We show that the lateral size increase in storage roots was accomplished through establishment of a singular vascular cambium and subsequent deposition of non-lignified parenchyma cells. Early storage root development was marked by production of lignified cells in the central cylinder. This coincided with the strong expression of important secondary cell wall biosynthesis genes, namely *UGDH*, *GAE*, *UXS*, and *GAUT*. The same genes were down-regulated in later stages where starch biosynthesis is the dominantly active pathway. Starch biosynthesis began only after the establishment of the vascular cambium, after which mainly xylem parenchyma cells were produced. We demonstrate that this was accompanied by strong transcriptional up-regulation of plastid-localized starch biosynthesis genes such as *PGM*, *AGPase*, *GBSS*, *SS*, and *BE*. The vascular cambium itself was formed through a transcriptionally strong auxin response correlated with expression of a *HD-ZIPIII* gene and subsequent expression of *PXY*, *CLE41*/*44*, and *WOX14*. The formation of parenchyma rather than sclerenchyma cells was accompanied by high expression of the *KNOX*/*BEL* genes *KNAT1*, *PNY*, and *PNF*, highlighting their importance for xylem parenchyma function.

While this study provides a groundwork for a lesser known crop species, it also raises many questions to be answered. For instance, how will direct genetic alteration of different developmental regulators alter storage root expansion and storage parenchyma formation? Understanding the role of *KNOX/BEL* factors and the role of different hormones in the suppression of secondary cell wall biosynthesis in storage parenchyma cells should yield useful information for further advances in cassava.

## Data Availability

The data for this study have been deposited in the European Nucleotide Archive (ENA) at EMBL-EBI under accession number PRJEB41121. The phylogenetic tree of *WOX* genes, RNA sequencing reads, KEGG pathway enrichment results, differentially expressed genes, and carbohydrate measurements are available at Dryad digital repository https://doi.org/10.5061/dryad.0cfxpnw0t; Rüscher *et al.* (2021)

## Abbreviations

AGL: *AGAMOUS-LIKE*
AGPase: *ADP-GLUCOSE PYROPHOSPHORYLASE*
AMY: *AMYLASE*
ANT: *AINTEGUMENTA*
AP1: *APETALA 1*
ARF: *AUXIN RESPONSE FACTOR*
AUX1: *AUXIN RESISTANT 1*
AUX/IAA: *AUXIN RESISTANT/INDOLE-3-ACETIC ACID INDUCIBLE*
AXS: *UDP-apiose/UDP-xylose synthase*
BE: *STARCH BRANCHING ENZYME*
BOP: *BLADE-ON-PETIOLE*
CLE: *CLAVATA3/ESR-RELATED*
CSLD: *CELLULOSE SYNTHASE-LIKE D*
cwINV: *CELL WALL INVERTASE*
CycD3;1: *CYCLIN D3;1*
DSR: developing storage root
GA2OX: *GIBBERELLIN-2-OXIDASE*
GA3OX: *GIBBERELLIN-3-OXIDASE*
GA20OX: *GIBBERELLIN-20-OXIDASE*
GAE: *UDP-glucuronic acid epimerase*
GBSS: *GRANULE-BOUND STARCH SYNTHASE*
GH3: *GRETCHEN HAGEN 3/INDOLE-3-ACETIC ACID-AMIDO SYNTHASE*
GID1: *GIBBERELLIN INSENSITIVE DWARF 1*
HD-ZIP III: *CLASS III HOMEODOMAIN LEUCINE ZIPPER TRANSCRIPTION FACTORS*
KNAT1: *KNOTTED-LIKE FROM ARABIDOPSIS THALIANA 1*
KS: *ENT-KAUREN SYNTHASE*
LBD: *LOB DOMAIN-CONTAINING PROTEIN*
NBR: non-bulked root
PHB: *PHABULOSA*
PGM: *PHOSPHOGLUCOMUTASE*
PHS: *STARCH PHOSPHORYLASE*
PMI: *PHOSPHOMANNOSE ISOMERASE*
PNF: *POUND-FOOLISH*
PNY: *PENNYWISE*
PTL: *PETAL LOSS*
PXY: *PHLOEM INTERCALATED WITH XYLEM*
SAUR: *SMALL AUXIN UPREGULATED RNA*
SS: *STARCH SYNTHASE*
SUS: *SUCROSE SYNTHASE*
TPS/TPP: *TREHALOSE-6-PHOSPHATE SYNTHASE/PHOSPHATASE*
UGDH: *UDP-GLUCOSE DEHYDROGENASE*
UGPase: *UDP-GLUCOSE PYROPHOSPHORYLASE*
UXS: *UDP-XYLOSE SYNTHASE*
vINV: *VACUOLAR INVERTASE*
WOX: *WUSCHEL-RELATED HOMEOBOX*
